# Examining Roles, Support, and Experiences of Community Health Workers During the COVID-19 Pandemic in Bangladesh: A Mixed Methods Study

**DOI:** 10.9745/GHSP-D-21-00761

**Published:** 2022-08-30

**Authors:** Shongkour Roy, Sarah Kennedy, Sharif Hossain, Charlotte E. Warren, Pooja Sripad

**Affiliations:** a Population Council, Dhaka, Bangladesh.; b Johns Hopkins Center for Communication Programs, Baltimore, MD, USA.; c Population Council, Washington, DC, USA.

## Abstract

**Introduction:**

Understanding community health workers’ (CHWs) experiences of sustaining routine health care promotion and provision activities as well as their challenges in adopting new responsibilities within a dynamic context is critical. This study explored the roles and perspectives of CHWs within the government-led coronavirus disease (COVID-19) community health response in Bangladesh.

**Methods:**

We conducted a mixed methods study to explore the government’s response to COVID-19 and its association with community health programming through a telephone-based survey of 370 government-employed CHWs. We also conducted 28 in-depth interviews with policy makers, program managers, CHW supervisors, and CHWs. We conducted exploratory and regression analysis of survey data and qualitative analysis of interview data.

**Results:**

The majority of CHWs reported receiving training related to COVID-19, including community-based prevention strategies from government and nongovernment stakeholders. Access to infection prevention supplies differed significantly by CHW cadre, and perspectives on the provision of adequate supplies varied qualitatively. CHWs reported slight decreases in routine work across all health areas early in the pandemic, and a majority reported added COVID-19-related responsibilities as the pandemic continued, including advising on signs/symptoms in their communities and referring suspected cases of COVID-19 for advanced facility care. Regression analyses showed that government support and integration of CHWs into their response—particularly being trained on COVID-19—predicted CHW capacity to advise communities on symptoms and provide routine services.

**Discussion:**

Government-employed CHWs in Bangladesh continued to provide health education and routine services in their communities despite pandemic- and response-related challenges. Varied support and differential CHW cadre-specific effects on COVID-19 awareness building in the community, referral, and routine service provision merit attention in Bangladesh’s pluralistic community health system. While COVID-19 infection and government-mandated lockdowns restricted CHW mobility, the workers’ capacity to continue service provision and education can be leveraged in vaccination and surveillance efforts moving forward.

## Abstract (precis)

Government-employed community health workers in Bangladesh are essential actors in the COVID-19 response in communities. Ensuring the workers’ equitable access to supportive mechanisms for their work, including training, infection prevention supplies, and supportive supervision, is critical for successfully preventing and managing COVID-19 in Bangladesh.

## Teaser

**Key Findings:**

- During the first wave of the coronavirus disease (COVID-19) pandemic in Bangladesh, across all health areas, community health workers (CHWs) described a slight decrease in the routine services they were able to provide due to restrictions in movement posed by lockdowns and other challenges.
- The government and various nongovernmental organizations provided supportive mechanisms to CHWs through training, supplies, and supportive supervision; however, these supports were not always uniformly distributed across cadres, leading to some discontent among CHWs.
- CHWs were crucial actors in the government’s COVID-19 response, as they took on new pandemic-related responsibilities in their communities to prevent the spread of the disease while continuing their routine work.

**Key Implications:**

- Government actors such as policy makers and nongovernmental actors involved in the community COVID-19 response in Bangladesh should consider CHW perspectives on supportive mechanisms and include them in future pandemic programming.
- National stakeholders can strive to ensure equitable support, incentives, and supervision to family welfare assistants and health assistants working across all health areas as they undertake new COVID-19 responsibilities in their communities.

## INTRODUCTION

### Community Health Workers in Pandemic Contexts

Community health workers (CHWs) are critical actors within community health systems globally, providing health education, promotion, and a range of reproductive, maternal, child, and primary health care services. As intermediaries between communities and health facilities, CHWs—health care workers with 6 months of initial training who provide care in community settings—often serve as the first points of contact for populations.^1^^,^^2^ A recent collection of articles demonstrate that CHWs often represent and engage communities actively in national policy implementation processes and tracking globally.^3^ As community members themselves, CHWs possess a unique understanding of the local context, including social barriers and facilitators to accessing timely and quality primary health care, and can facilitate effective linkages to care. In the context of disease outbreaks, such as the current global COVID-19 pandemic, CHWs continue to serve in this integral role as trustworthy sources of health information, prevention, and care, often performing their routine duties with added responsibilities.^4^^–^^6^

In humanitarian crises, including disease outbreaks that expose shortcomings of any health system, protecting health workers (including CHWs) so that they feel safe and supported in their work is critical; providing personal protective equipment (PPE), logistics, supportive supervision, and mental health support are ways in which governments and nongovernmental organizations (NGOs) can ensure support and safety.^7^^,^^8^ While facility-based provider perspectives have been somewhat studied in humanitarian crises, with an emphasis on technical abilities, basic clinical and communications skills, and resources and guidelines availability,^9^ studies have rarely considered CHWs and their perspectives.^10^^,^^11^ A rapid evidence review of CHWs’ performance in pandemic contexts (severe acute respiratory syndrome, Middle East respiratory syndrome, Ebola, and Zika) suggests that CHWs are often drawn into roles that increasingly require them to function at elevated skill levels and add to their routine activities (e.g., contact tracing, monitoring care for new diseases).^4^ With the emergence of the global COVID-19 pandemic, CHWs have been at the forefront of the response.

In pandemic contexts, CHWs are often drawn into roles that require them to function at elevated skill levels and add to their routine activities.

### Government-Employed CHWs in Bangladesh

Bangladesh has a variety of cadres of CHWs, employed by the government or NGOs, working across a variety of health areas. The current study focused on 2 cadres of government-employed CHWs—family welfare assistants (FWAs) and health assistants (HAs). FWAs provide counseling and promotion of family planning (FP) services in communities and clinics under the Directorate General of Family Planning, while HAs support the Expanded Programme on Immunization (EPI) and disease surveillance and provide other primary health care services under the Directorate General of Health Services. While similarities and differences in FWA and HA roles exist, both cadres have overlapping responsibilities in service provision to the community (Figure). Moreover, both cadres are expected to provide services at least 2 days per week in fixed facilities called community clinics.^5^ FWAs directly report to FP inspectors and HAs directly report to assistant health inspectors/health inspectors. Both FWAs and HAs are expected to check in at least monthly with their supervisors.

**FIGURE:**
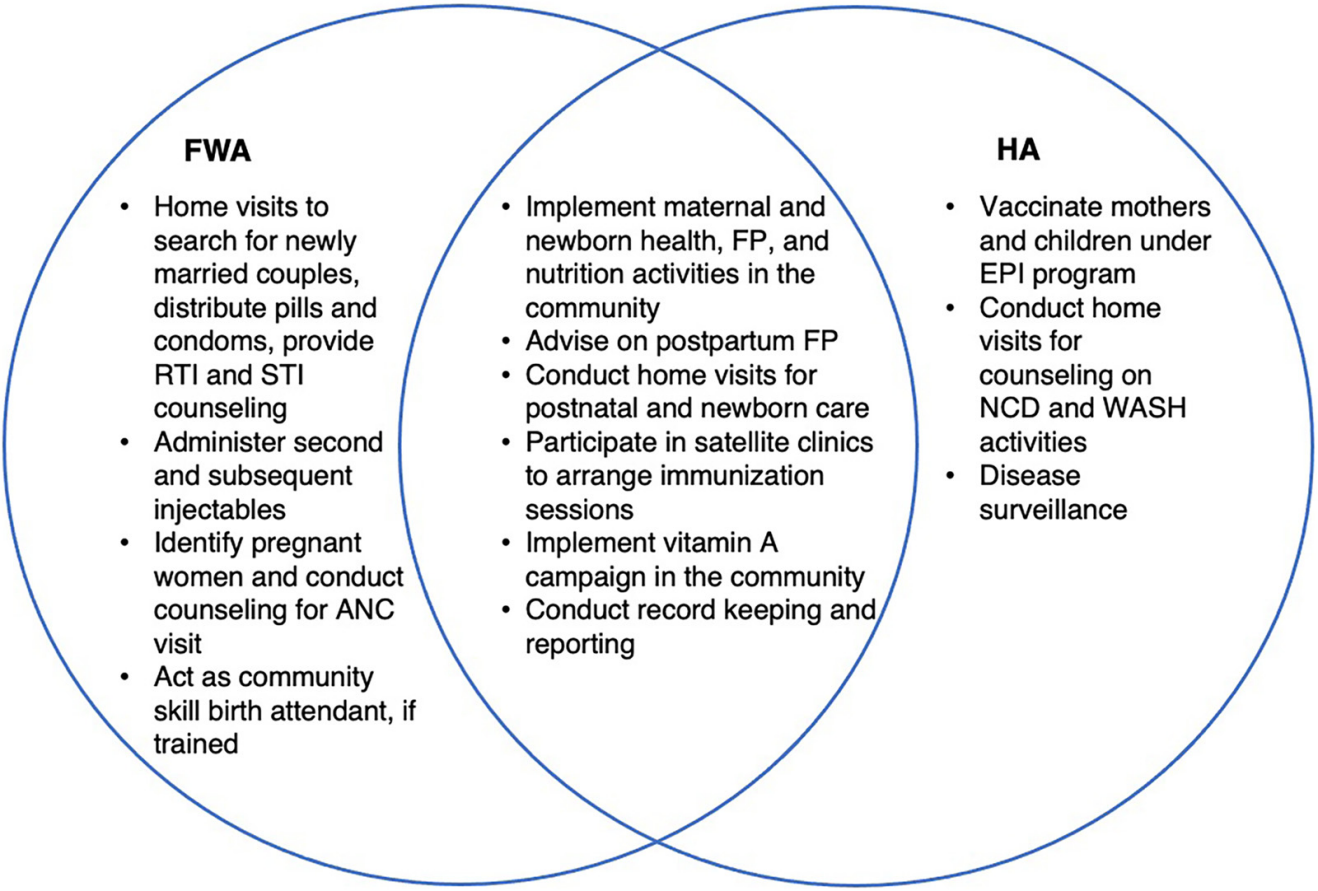
Comparison of Responsibilities Between FWAs and HAs in Bangladesh Abbreviations: ANC, antenatal care; EPI, Expanded Programme on Immunization; FP, family planning; FWA, family welfare assistant; HA, health assistant; NCD, noncommunicable disease; RTI, reproductive tract infection; STI, sexually transmitted infection; WASH, water, sanitation, and hygiene.

### COVID-19 in Bangladesh and the Government’s Community Health Care Response

COVID-19 was first detected in Bangladesh on March 8, 2020, in the capital city, Dhaka, and within a month, cases were reported in all divisions of the country.^12^ From April to May 2020, the number of reported infections continued to climb, before reaching a peak in June. Thereafter, from July to November 2020, cases dropped. This period of March to November 2020 is considered the first wave of the pandemic in Bangladesh.^12^ During the first wave, the government of Bangladesh implemented a 10-day lockdown on March 26, 2020, which was gradually extended through May 31, 2020. As of November 17, 2021, there were 1,572,948 confirmed cases and 27,928 confirmed deaths in the country.^13^

In July 2020, with technical guidance from the Ministry of Health and Family Welfare, the government of Bangladesh released the Preparedness and Response Plan for COVID-19.^14^ The plan aimed to prevent and control the spread of COVID-19 in Bangladesh to reduce its impact on the population’s health, its well-being, and the economy of the country, as well as to establish the framework to treat those infected. Strategies included implementing periodic countrywide or district/area lockdowns, compulsory mask wearing and social distancing in public, promoting safe hygiene practices, enforcing isolation/quarantine for suspected/confirmed cases, facilitating institutional treatment for people with severe symptoms, and facilitating community-based prevention practices by engaging CHWs to respond to COVID-19 in their communities.^14^

During lockdown periods, the government halted all “nonessential” businesses and activities, which affected institutions such as schools, mosques, offices, some factories, and most local shops/businesses. Essential personnel included health workers (such as CHWs), domestic workers, essential factory workers (garment industry), and government employees.^14^ As a result of general restricted mobility, CHWs were constrained by lockdowns, and the use of community and facility-based services was affected.^15^ CHWs’ home visits were slightly disrupted or had to be altered. Furthermore, increased workloads due to health workers falling ill from COVID-19 exacerbated an existing shortage of CHWs and affected workers’ ability to distribute services equitably.^16^ Beyond community health, mothers in need of institutional delivery services faced higher disruption because many hospital authorities asked patients to bring certificates indicating they were not infected with COVID-19, which created anxiety and led to reduced care-seeking for antenatal care, institutional delivery, and postnatal care.^15^^,^^17^ COVID-19 also affected essential facility-based health services beyond reproductive, maternal, newborn, and child health.^15^ A lack of medicine, hospital beds, and doctors and nurses impeded health care service access. Because of these challenges, many non-COVID-19 patients with noncommunicable diseases that required urgent medical intervention or surgery, such as cancer, cardiovascular disease, and kidney disease, received delayed care from hospitals.^18^

Given CHWs’ importance in providing community health education and essential health care services, further investigation is required to situate their experiences and unpack the value of community health under these circumstances. Our objective in this study was to explore how COVID-19 affected the roles and experiences of FWAs and HAs in 4 geographically diverse districts in Bangladesh.

We aimed to explore how COVID-19 affected CHWs’ roles and experiences in Bangladesh.

## METHODS

### Study Design

We used a mixed methods design to examine the government’s community health response to COVID-19 and its overall impact on CHWs’ roles and experiences in Bangladesh. A semistructured telephone-administered survey with FWAs and HAs was used to investigate their routine and additional responsibilities in the COVID-19 context, the support they were provided, and the challenges they experienced. Telephone-administered in-depth interviews (IDIs) at the national, subnational, and community levels were conducted with policy and program stakeholders including FP inspectors, assistant health inspectors, health inspectors, upazila FP officers, upazila health and FP officers, the deputy director of FP, FWAs, and HAs. IDIs explored the government of Bangladesh’s community health COVID-19 response, including the implementation of guidelines, support provided to both cadres of CHWs, and formal or spontaneous coordination between various governmental and nongovernmental stakeholders.

### Study Setting and Participants

This study was nested within Population Council’s research agenda under the Frontline Health project (2018–2021), which explored the perspectives of CHWs and other stakeholders during the pandemic on a range of issues, including quality and experience of services provided by CHWs in various health areas, among other operational and contextual considerations affecting the institutionalization of community health across 7 priority low- and middle-income countries.^19^ This study was conducted in 4 geographically diverse districts of Bangladesh—Khulna, Rajshahi, Sylhet, and Cox’s Bazar—including within a humanitarian setting where Rohingya refugees were temporarily living (Cox’s Bazar). All study sites had active government-employed CHWs providing health services and reflected ongoing Frontline Health project work.

With one of the oldest CHW programs globally, which began in the 1970s, Bangladesh has several well-established cadres of CHWs employed by both the government^16^ and NGOs, for example, the Bangladesh Rural Advancement Committee.^17^ However, this study did not include CHWs employed by NGOs and only 2 cadres of government-employed CHWs were interviewed for this study.

Participants in this study also included policy makers or program managers at the national, upazila, and local supervisory/inspector levels in both FP and the general maternal and child health space. These stakeholders were selected based on their involvement in government-implemented community health policy and programming in Bangladesh.

### Recruitment and Data Collection

Data collection took place between November and December 2020. Before data collection, the study team collated a list of more than 450 government CHWs with names and phone numbers in the 4 study areas based on a pool of CHWs previously recruited and studied under the Frontline Health project.^20^

We estimated a random sample size of 443 CHWs based on a sample size calculation for exploratory studies that assumes a 95% confidence interval and a 5% margin of error. We assumed 75% of CHWs were involved in the COVID-19 government response (*P*=.75) and a 35% nonresponse adjustment for the use of the phone-based survey. The respondents for the quantitative survey and IDIs were selected purposively because of their availability and willingness to participate in the phone survey and prior experience in community health. The study team used a previously determined list of CHWs’ and community health policy and program stakeholders’ telephone numbers who granted permission for future contact. For the qualitative IDIs, additional participants were recruited based on their experience and understanding of roles involved in the COVID-19 response. The study coordinator and team applied a 2-step call authentication process to recruit and interview participants. After an initial call to explain the study purpose and gauge potential interest, the study team scheduled a date/time to administer the survey with each CHW. A final sample of 370 CHWs (204 FWAs and 166 HAs) participated in the quantitative survey. Qualitative IDIs were conducted with 20 policy makers, program managers, and CHWs’ immediate supervisors and with 8 CHWs (28 IDIs in total). The survey and IDIs were administered concurrently during the study period in the Bengali language by trained data collectors using semistructured study instruments, which included a questionnaire and an IDI guide, respectively.

### Data Analysis

Our mixed methods analysis drew on complementary information collected quantitatively and qualitatively to explore national and subnational responses to the COVID-19 pandemic (including how the pandemic affected community health at large), and the roles, supports, and challenges of CHWs interacting with their communities and health facilities in the context of COVID-19. The methods were triangulated at the final analysis phase to answer the research questions.

This mixed methods analysis included exploration of the roles, supports, and challenges of CHWs interacting with their communities and health facilities in the context of COVID-19.

Quantitative data were cleaned and analyzed using Stata 14 (StataCorp). We first described the characteristics of recruited CHWs (differentiating between FWAs and HAs), including gender, age, education, duration of work experience, incentives received, distance from worksite, and mode of transportation. A bivariate analysis followed to explore the differences between demographic characteristics of FWAs and HAs (Table 1), support they received during the pandemic (e.g., COVID-19-related training, prevention, safety materials, supportive supervision), their routine responsibilities (e.g., several health areas) before and during the pandemic, and their new responsibilities (e.g., educating people, advising signs/symptoms of COVID-19 and transmissibility, and referring suspected case testing, quarantine/isolation, and facility care). Finally, we applied a multivariate logistic regression model to identify which support factors were associated with routine and the added service provision outcomes. This final analysis used dichotomized scores of summary indices composed of binary (yes/no) indicators for CHWs advising signs/symptoms of COVID-19 (8 items, Table 2), advising community how COVID-19 can be transmitted (6 items, Table 2), and/or advising community how to prevent COVID-19 (6 items, Table 2). If CHWs scored ≥90%, we considered them “correctly advised” versus “incorrectly advised.” A summary index for training, sufficient supplies, and supportive supervision that scored ≥90% was similarly constructed (Table 3). Referral of suspected COVID-19 cases was measured by a single binary question: “Are CHWs referring suspected COVID-19 cases for advanced care at facilities?” Similarly, routine service provision was measured using a single question: “Have you continued carrying out your routine responsibilities?” Individuals who responded “somewhat” or “yes” were grouped into a category for comparison with those who were not able to continue their routine responsibilities.

**TABLE 1.** Demographic Characteristics of Community Health Workers in Bangladesh

	FWA, % (n=204)	HA, % (n=166)	Total, % (N=370)	*P* Value
Gender				
Female	100	56.6	80.5	<.001
Male	0	43.4	19.5	
Age, years				
<30	10.8	1.8	6.8	<.001
30–35	20.1	17.5	18.9	
36–40	3.9	21.1	11.6	
41–45	5.4	6.0	5.7	
46–50	19.6	25.3	22.2	
>50	40.2	28.3	34.8	
Education level				
Secondary (incomplete)	2.9	0.0	1.6	<.001
Secondary (complete)	41.7	0.6	23.2	
Higher secondary and above	55.4	99.4	75.2	
Time working as a CHW, years				
<5	8.8	0.6	5.1	<.001
5–10	13.3	4.8	9.5	
>10	77.9	94.6	85.4	
Received incentives^a^				
Salary	100	100	100	NA
Event-related allowances (transport, lunch, airtime)	80.4	84.3	82.2	.32
Event-related nonfinancial (food, backpack)	70.1	81.9	75.4	.01
Location of work				
Urban informal settlements	11.8	1.8	7.3	<.001
Peri-urban	9.8	31.3	19.5	
Rural	78.4	66.9	73.2	
Time to reach farthest household for CHWs that live in community,^b^ minutes				
≤10	26.6	7.0	20.1	<.001
11–20	40.8	41.2	40.9	
21–30	23.1	34.1	26.8	
>30	9.5	17.7	12.2	
Main mode of travel since pandemic				
By foot	23.0	3.6	14.3	<.001
Bicycle	0.0	1.8	0.8	
Motorbike	6.9	21.1	13.3	
Bus	0.5	3.0	1.6	
Taxi/compressed natural gas taxi	30.9	40.4	35.2	
Easy bike/rickshaw/boat	38.7	30.1	34.8	
Geographic working area				
Rajshahi	24.0	31.3	27.2	.01
Khulna	23.5	18.7	21.4	
Sylhet	26.0	38.0	31.4	
Cox’s Bazar	26.5	12.0	20.0	

Abbreviations: CHW, community health worker; FWA, family welfare assistant; HA, health assistant; NA, not applicable.

a Multiple responses.

b N=254.

**TABLE 2.** CHWs in Bangladesh Who Reported Undertaking Added Responsibilities Related to COVID-19 Prevention, Treatment, Referring, and Reporting

	FWA, % (n=204)	HA, % (n=166)	Total, % (N=370)	*P* Value
Educating community members about COVID-19 prevention/treatment	98.5	100	99.2	.11
Advising about signs/symptoms of COVID-19^a^				
Fever	98.5	96.9	97.8	.32
Dry cough	91.0	89.8	90.5	.67
Sore throat	58.7	62.7	60.5	.44
Nasal congestion	68.2	76.5	71.9	.07
Sneezing	39.3	25.3	32.9	.004
Headache	23.8	19.8	22.1	.35
Breathing difficulties	51.7	45.2	48.8	.21
Loss of taste	11.4	15.1	13.1	.30
Correctly advising signs/symptoms of COVID-19 (score≥90%)	25.5	20.5	23.2	.25
Advising community how COVID-19 can be transmitted^a^				
Coughing/sneezing by infected people	91.0	97.6	94.0	.008
Direct contact with infected people	74.6	82.5	78.2	.06
Being close to infected people	61.2	64.5	62.7	.52
Touching contaminated objects/surfaces/ disposable masks	30.4	29.5	29.9	.86
Correctly advising community how COVID-19 can be transmitted (score≥90%)	9.3	15.7	12.2	.06
Advising community how to prevent COVID-19				
Stay at home	96.0	98.8	97.3	.10
Frequent hand washing	100	100	100	NA
Wearing mask	100	100	100	NA
Social distance	100	98.8	99.5	.11
Quarantine if exposure is suspected	97.0	99.4	98.1	.09
Self-isolate	99.5	100.0	99.7	.36
Correctly advising community how to prevent COVID-19 (score≥90%)	92.7	98.2	95.1	.02
Referring suspected COVID-19 cases for advanced care at facilities	56.4	84.3	68.9	<.001

Abbreviations: CHW, community health worker; COVID-19, coronavirus disease; FWA, family welfare assistant; HA, health assistant; NA, not applicable.

a N=367 (FWA, n=201; HA, n=166).

**TABLE 3.** COVID-19-Specific Training, Supplies, and Supportive Supervision Received by Community Health Workers in Bangladesh

	FWA, % (n=204)	HA, % (n=166)	Total, % (N=370)	*P* Value
COVID-19-related training provided to CHWs	65.7	97.6	80.0	<.001
Types of training^a^				
Preventive strategies (handwashing practices, social distance, self-quarantine, etc.)	100	100	100	NA
General information about COVID-19	100	100	100	NA
Correct use of PPE (masks, gloves, apron, etc.)	97.8	98.8	98.3	.50
Signs and symptoms of COVID-19	98.5	100	99.3	.11
Contact tracing and community surveillance	18.7	88.3	56.8	<.001
Home-based care of COVID-19 cases	80.6	87.7	84.5	.09
Continuity of community-based services	67.2	77.8	72.9	.04
Supplies provided to CHWs				
Disposable masks	77.9	88.6	82.7	.007
Reusable masks	63.2	32.5	49.5	<.001
Hand sanitizer	96.1	88.5	92.7	.005
Gloves	97.1	86.1	92.2	<.001
Soap	8.3	69.9	35.9	<.001
Disinfect supplies	24.0	40.4	31.4	.008
Job aids for COVID-19	15.2	45.8	28.9	.001
Sufficient supplies (score≥90%)	5.9	18.1	11.4	.001
Methods of supportive supervision to CHWs				
Regular in-person meeting	31.9	4.8	19.7	<.001
Scheduled team meeting (via phone)	97.1	97.6	97.3	.7
Digital communication (WhatsApp, text, etc.)	46.6	16.3	32.9	<.001
Ad hoc phone call	89.2	92.8	90.8	.2
Supportive supervision (score≥90%)	23.5	1.2	13.5	<.001

Abbreviations: CHW, community health worker; COVID-19, coronavirus disease; FWA, family welfare assistant; HA, health assistant.

a N=296 (FWA, n=134; HA, n=162).

The qualitative IDIs explored complementary themes to unpack policy and management perspectives on the pandemic, including perspectives on governmental and nongovernmental responses and how the pandemic affected CHWs’ work. Qualitative data were audio recorded, transcribed in Bengali, and translated into English. The IDI transcripts were then coded and analyzed using QSR International’s NVivo 12 software. A thematic content analysis technique was applied using an inductively derived coding framework developed through an iterative process. A team of 2 researchers read through the transcripts and identified emerging themes to include in the coding framework, which they discussed and finalized with 2 other study team members. Themes included the impact of COVID-19 on community health, including how various governmental and nongovernmental actors responded and provided support to CHWs through training, supplies, and supervision; the impact of COVID-19 on CHWs’ routine work and their added responsibilities; and the challenges experienced by CHWs in the context of the pandemic. The study team reviewed the codes together and corroborated them with key quantitative findings to respond to the overall objectives.

### Ethical Approval

Ethical approval for this study was provided by the Population Council’s Institutional Review Board (Protocol 942) and Bangladesh Medical Research Council (#32228072020).

## RESULTS

We describe the findings in 2 main sections: (1) the national response to COVID-19 at the community level and (2) the association between COVID-19 and CHWs’ workload, including their increased roles and responsibilities, and their challenges in the context of the pandemic.

### Demographic Characteristics of CHWs

The majority of the 370 CHWs in our sample were female (80.5%), more than a third were aged older than 50 years (34.8%), and over three-quarters (75.2%) completed higher secondary education or above (Table 1). Most CHWs reported that they had been working for more than 10 years (85%), and more than 60% were able to reach their clients in 20 minutes or less.

### National Responses: Training, Supplies, and Supportive Supervision for CHWs

We describe the CHWs’ experiences of the government response to the pandemic, specifically pertaining to supportive mechanisms such as training, the provision of supplies, and supportive supervision.

#### Training Provided to CHWs

The majority (80%) of CHWs reported receiving COVID-19-related training from the government during the pandemic, but significantly more HAs than FWAs reported receiving such training (at the time of the survey). HAs also received significantly more training than FWAs on contact tracing and community surveillance and continuity of community-based services. HAs were more actively engaged in contact tracing and community surveillance based on their broader scope of experience in prior infectious disease prevention (Table 3).

The majority of CHWs reported receiving COVID-19-related training during the pandemic, but significantly more HAs than FWAs reported receiving such training.

Qualitative and quantitative data were aligned on training. All CHWs interviewed described receiving some training/guidance on COVID-19 from the government and/or collaborating NGOs, although some noted that training was optional or delayed for months. Across study sites, training focused on increasing awareness of signs and symptoms of COVID-19, the importance of protection measures (e.g., mask wearing, maintaining social distance, using sanitizer), and the need to seek testing and advanced care if severe symptoms arose. In Cox’s Bazar, waste management training was also included as a component. In a few cases during the initial phase of the pandemic, material resources including job aids were provided even if no formal in-person training occurred because of lockdown restrictions.

*We got to know about it [COVID-19] from social media. About a month and half ago we had an optional training, but at the beginning, the office provided us charts. All the symptoms and precautions were mentioned in different charts. At the [onset] of the pandemic, it was not permitted to meet, so the office provided us equipment and charts, there was no training [at onset]. We learned these from social media, television, and charts all by ourselves.* —FWA, Cox’s Bazar

#### Supplies Provided to CHWs

The majority of CHWs reported that they received some infection prevention supplies such as disposable or reusable masks and gloves; however, HAs reported significantly higher receipt of job aids, disinfecting supplies, and soap, while FWAs reported significantly higher provision of gloves, hand sanitizer, and reusable masks (Table 3).

Qualitatively, some contradictory perspectives were apparent among CHWs and program stakeholders regarding the supplies that CHWs received for their work. While program stakeholders felt sufficient PPE supplies (e.g., masks, gloves, and hand sanitizer) were distributed, some CHWs felt that the provision of supplies was inadequate; other CHWs in different districts reported not receiving any supplies at all.

*We have given them PPE, masks, hand sanitizer, gloves, and also the head cover to ensure that they are not infected and can provide the services.* —Upazila FP officer, Sylhet

*Now, the government has given us lots of PPE and I have distributed them among the HAs, assistant health inspectors, and health inspectors. Even my office staff have the PPE. The government has given us sanitizer, masks, and PPE, and NGOs give us logistics.* —Upazila health and FP officer, Khulna

*We did not get masks, gloves, PPE, let alone extra supply of these. They did not give us the necessary equipment needed to work. We buy and use these by ourselves. Nothing is provided by our office.* —HA, Rajshahi

Community health program and policy stakeholders indicated that, in addition to the government-steered response to COVID-19, NGOs play a crucial role in supporting CHWs and the community health system in Bangladesh. Program managers felt that NGO support empowers CHWs to carry out their work by supplementing training, supplies, and PPE.

*NGOs are also helping us by supplying PPE and masks. Another NGO has given us 2 ICU beds. So, all of them have given us enough support. Because of these factors, we feel the courage to work.* —Upazila health and FP officer, Rajshahi

#### Supervision Provided to CHWs

Most CHWs reported receiving some form of supportive supervision during the pandemic, with scheduled team meetings and ad hoc phone calls being the most common methods for both FWAs and HAs. Notably, significantly more FWAs than HAs were supervised via digital communications and regular in-person meetings (Table 3).

Most CHWs reported receiving some form of supportive supervision during the pandemic.

Similarly, CHW descriptions of “supervision” qualitatively varied from their supervisors; while supervisors emphasized the flexibility in the ways they supervised, FWAs and HAs preferred in-person meetings for better communication. Supervisors described being available to support and advise CHWs via mobile phones (as opposed to conducting meetings in person); for larger issues, they were able to meet the CHWs in person.

*CHWs always communicate with our office via phone. But training should be conducted through physical meetings. That would be better.* —FWA, Rajshahi

*For communication, we used mobile phones. But those problems which couldn‘t be solved over the mobile phone, they were coming with that. There was no effect of [COVID-19] over that. We ordered them not to come to the office or connect to the internet unless you have a big problem.* —Upazila FP officer, Rajshahi

*Actually, at that time, we were mainly communicating virtually. We talked with them [CHWs] over the telephone or arranged meetings by using Zoom or Google Meet. We continued our communication through this medium as there was a social distance issue. So, there were different types of challenges, and we overcame them. In this digital Bangladesh, it is possible to work from home. —*Upazila FP officer, Sylhet

### Effects of COVID-19 on CHWs’ Routine Service Provision

The government of Bangladesh prohibited most people from leaving their homes during COVID-19 lockdowns except for essential/technical workers, including health workers; therefore, FWAs and HAs continued to provide health services in their communities and facilities. Community health service delivery was never officially or completely “stopped,” except for group meetings held by CHWs.

Community health service delivery was never officially or completely “stopped” during COVID-19, except for group meetings held by CHWs.

#### Continuity of Service Provision and Adaptations to How CHWs Work

In the CHW survey, respondents were asked to recall their frequency of service provision both before and during the pandemic. During the pandemic, across all health areas, CHWs described disruptions in the routine services they were able to provide during the first wave, such as home-based services for FP and home-based care for postpartum women and newborns (Table 4). CHW routine responsibilities were also disrupted during COVID-19; immunizations for children decreased 6.5% and home-based services for FP decreased 4.1% (data not shown).

**TABLE 4.** CHWs in Bangladesh Who Reported Undertaking These Activities Before^a^ and During the COVID-19 Pandemic

	FWA, % (n=204)		HA, % (n=166)		Total, % (N=370)		*P* Value
	Before	During	Before	During	Before	During		
Home-based services for family planning	100	92.7	11.5	11.4	60.3	56.2	.38
Home-based visits for infectious disease prevention and management	53.4	41.7	99.4	98.8	74.1	67.3	.08
Home-based care for postpartum women and newborns	99.5	93.6	44.6	44.4	74.9	71.6	.38
Mental health counseling	91.2	81.3	75.3	72.9	77.3	73.2	.26
Immunizations for children	78.9	71.5	100	99.4	84.1	77.6	.04
Nutrition supplementation	97.6	89.2	99.4	97.5	88.4	84.1	.11
Make referrals to a health facility	98.5	90.2	100	99.4	98.4	92.9	.001
Discuss safe drinking water and/or handwashing	99.5	98	100	100	99.1	94.3	.001
Counseling and direct primary health service	100	92.6	100	99.4	100	98.9	.09

Abbreviations: CHW, community health worker; COVID-19, coronavirus disease; FWA, family welfare assistant; HA, health assistant.

a Before=before March 2020; during=March 2020 to November 2020.

Qualitative findings reiterate that most CHWs continued to provide routine services at relatively high levels, but the lockdown prevented some FWAs and HAs from delivering community-based services because of restrictions in movement (preventing household visits), the shutdown of public transportation and nonessential businesses, and communities’ refusal to see them.

*The lockdown started on 26th March 2020. So, there was no system to go from one house to another. The strict situation continued till April. Then, gradually it became normal.* —FP inspector, Sylhet

*It has become difficult to go to people’s houses for vaccination…We faced trouble around communication because there was lack of transportation, and we were stopped from going to our destination…even after showing our identity card. We worked under more difficulties than the normal time.* —HA, Rajshahi

FWAs specifically described continued provision of FP counseling to households; however, they were no longer able to conduct large health education or counseling sessions such as courtyard meetings as they did before the pandemic. They did not enter households unless they were allowed in; rather, they often met clients outside their homes to maintain social distancing.

*We were really frightened for first 3 months. We could do nothing but distribute some general contraceptives like pills or condoms. But now we are doing just fine. We are freely working with other contraceptive methods too.* —FWA, Rajshahi

*Courtyard meetings were canceled. There was less use of injection method as some of our clients were afraid to come. Women took [tetanus toxoid] vaccine less than usual.* —FWA, Sylhet

In terms of providing guidance and troubleshooting problems in their communities, many CHWs relied on mobile phones to talk to their clients in the context of the pandemic, as opposed to traditional face-to-face interactions. CHWs continued to work at facilities such as community clinics and to provide direct services to clients (with safety measures implemented) such as the provision of medicine, vaccines, and injectable contraception.

*We can’t talk to our clients directly. In the past, we entered their house and counseled them properly. But we cannot do that anymore. We have advised them over phone.* —FWA, Khulna

#### CHWs’ Added Pandemic Responsibilities

In addition to carrying out their routine responsibilities, CHWs reported engaging in new responsibilities related to COVID-19 prevention, treatment, referring, and reporting. Almost all CHWs reported educating community members about COVID-19 prevention or treatment (Table 2). Both cadres of CHWs also reported advising about specific signs or symptoms of COVID-19, and how to prevent COVID-19 in their communities (Table 2). However, around a quarter or less of both cadres were not correctly advising on signs and symptoms of COVID-19 or how it was transmitted because of inadequate training, and they did not receive sufficient aid for providing information. Significantly more HAs than FWAs referred suspected COVID-19 cases for advanced care at facilities.

In addition to carrying out their routine responsibilities, CHWs reported engaging in new responsibilities related to COVID-19 prevention, treatment, referring, and reporting.

Qualitative findings describe that CHW responsibilities increased during the pandemic as they responded to COVID-19 in their communities. CHWs described their additional tasks, including educating communities on COVID-19 prevention or treatment (and misinformation related to COVID-19), how to take care of someone with COVID-19 in the home, referring suspected cases for testing, and reporting suspected or confirmed cases.

*When we see someone is showing corona symptoms, we advise them to take a test. And we try to keep updated with their condition like asking them if they have taken the test or not. Sometimes they don’t as they think these all are just common cold symptoms. We try to make them maintain social distancing when they come to us for health services. Ask them to wash their hands for 20 seconds at least.* —FWA, Rajshahi

The new COVID-19 responsibilities for CHWs increased their workloads, exacerbating an existing problem of a CHW shortage in Bangladesh. CHWs qualitatively described the burden of high workloads from the addition of COVID-19 responsibilities to their routine work. Some CHWs voiced their discontent with a lack of incentives to perform additional work.

*This is a problem for me as this is an extra burden over my regular job. I have to provide support for COVID-19 patients, to make and submit reports about them, providing medicines by going to their houses. We have to do our routine duties like visiting clinics, doing EPI, visiting the field, which is already a burden to us. Then comes this burden of extra work. But there is nothing in exchange. We have pressure of work and there is nothing in exchange.* —HA, Rajshahi

#### Factors Associated With CHW Routine Work and Added Responsibilities

Using the multivariate models, we explored the association of training, supplies, and supportive supervision with several roles of CHWs during the pandemic (advising on signs/symptoms of COVID-19 and on transmission, referrals, and provision of routine services) (Table 5). The model results suggest that CHWs who received COVID-19 training were 6.49 times as likely to advise on signs/symptoms of COVID-19 (aOR=6.49; 95% CI=2.58, 16.28) and 6.16 times as likely to provide routine services (aOR=6.16; 95% CI=3.22, 11.81) as those who did not receive training. Supportive supervision was significantly associated with referring suspected COVID-19 cases for advanced care at facilities (aOR=5.7; 95% CI=2.46, 13.20) but not associated with other predicted variables. Supplies status was not significantly associated with any service provision indicators in the adjusted models. HAs were significantly less likely than FWAs to educate on signs/symptoms of COVID (aOR=0.44; 95% CI=0.23, 0.87); however, they were 8.34 times as likely to refer suspected COVID-19 cases for further care at facilities (aOR=8.34; 95% CI=4.25, 16.4) as FWAs.

**TABLE 5.** Multivariate Relationship Between Service Provision in Pandemic With CHWs in Bangladesh Who Received COVID-19-Related Training, Supportive Supervision, and Supplies^a^

	Model 1		Model 2		Model 3		Model 4	
Indicators	Advising Signs/ Symptoms of COVID-19		Advising Community How COVID-19 Can Be Transmitted		Referring Suspected COVID-19 Cases for Advanced Care at Facilities		Providing Routine Services	
	OR(95% CI)	aOR (95% CI)	OR(95% CI)	aOR (95% CI)	OR(95% CI)	aOR (95% CI)	OR(95% CI)	aOR (95% CI)
Type of CHWs, HA (ref=FWA)	0.75 (0.46, 1.23)	0.44^b^ (0.23, 0.87)	1.80 (0.96, 3.39)	1.14 (.048, 2.71)	4.16^c^ (2.52, 6.88)	8.34^c^ (4.25, 16.4)	2.34^c^ (1.51, 3.61)	0.74 (0.38, 1.41)
Received COVID-19 training (ref=no)	4.19^b^ (1.75, 10.1)	6.49^c^ (2.58, 16.28)	2.81 (0.97, 8.12)	1.93 (0.61, 6.05)	1.25 (0.73, 2.15)	0.61 (0.32, 1.13)	6.62^c^ (3.71, 11.80)	6.16^c^ (3.22, 11.81)
Supportive supervision (ref=no)	0.80 (0.38, 1.68)	0.56 (0.25, 1.28)	0.98 (0.39, 2.45)	1.30 (0.46, 3.64)	2.64^b^ (1.19, 5.81)	5.7^c^ (2.46, 13.20)	0.33^b^ (0.18, 0.62)	0.32^b^ (0.15, 0.67)
Supplies status (ref=insufficient)	1.13 (0.48, 2.20)	1.26 (0.56, 2.80)	1.52 (0.63, 3.67)	1.36 (0.54, 3.42)	2.98^b^ (1.22, 7.30)	1.54 (0.60, 3.95)	1.94 (0.95, 4.0)	2.14 (0.95, 4.80)

Abbreviations: aOR, adjusted odds ratio; CHW, community health worker; CI, confidence interval; COVID-19, coronavirus disease; FWA, family welfare assistant; HA, health assistant; OR, odds ratio.

a Models are adjusted with CHWs’ working experience and level of education (primary, secondary, etc.).

b *P*<.01.

c *P*<.001.

CHWs reported various challenges with supplies as well as health and safety-related challenges while carrying out their work during the pandemic. The biggest challenge was fear of contact with infected people (49.4% of FWAs, 42% of HAs; data not shown), not having enough medicines in facilities, hospitals being overwhelmed by patients with COVID-19, and transport to facilities being unavailable. CHWs reported various reasons for not continuing routine work in the initial months of the pandemic, including a lack of guidelines. Significant differences were found in guideline awareness between FWAs and HAs (22.5% of FWAs, 4.3% of HAs; *P*<.001).

Qualitatively, CHWs and their program managers reiterated that CHWs’ fears of becoming infected and COVID-19 positivity rates among health workers led to fewer staff being available to provide services.

*As the number of our workers is fixed, so they become positive. When a worker in the clinic is infected, we need to leave him/her for 14 days. It becomes difficult to run the center at that time. It causes a problem for the local people as well. —*Upazila health and FP officer, Rajshahi

*There was positive case of COVID-19 in a block. The environment of the area was awful Then there was an EPI session, and the child of the patient was bought to the EPI center for vaccination. I told the mother to come in the next session of EPI. We were panicking at that time that we will be affected by the virus within no time. —*HA, Cox’s Bazar

## DISCUSSION

Our study findings suggest that government-employed CHWs in Bangladesh, specifically FWAs and HAs, have been essential health actors in the COVID-19 response. Despite significant challenges posed by the COVID-19 pandemic, FWAs and HAs continued providing vital health education and services in their communities. The government’s COVID-19 response in Bangladesh engaged CHWs through mandated guidelines around safety, hygiene, and virus protection, leveraging existing supervisory chains and coordinating efforts with the NGO sector regarding awareness building/training, supplies, and supervision. The government and NGO sectors (public and private) collaborated in various efforts, including CHW distribution of masks and in creating CHW-conveyed messages about COVID-19, including how to prevent it and what to do if exposed. Additionally, CHWs working for the NGO sector were essential in contributing to the surveillance and screening for symptoms of COVID-19.^21^ However, pandemic-related challenges had varying effects on the roles and experiences of FWAs and HAs and geographical regions, particularly Cox’s Bazar. Although COVID-19 similarly affected regions in Bangladesh, CHWs working in Cox’s Bazar felt a greater burden or workload because of refugee camp conditions, including close proximity and limited water, sanitation, and hygiene.

Despite significant challenges posed by the COVID-19 pandemic, FWAs and HAs in Bangladesh continued providing vital health education and services in their communities.

In the Bangladesh government’s COVID-19 response, both HAs and FWAs have similar responsibilities for providing general health education, including conveying information to communities about signs and symptoms, sensitizing people on the correct use of masks, and communicating various prevention strategies such as handwashing practices, social distancing, and self-quarantining to their communities. HAs were more involved in contract tracing and community surveillance than FWAs. As such, although nearly all CHWs reported educating community members about COVID-19, fewer referred suspected positive cases. Increased referrals were associated with being an HA (compared with FWA) and supportive supervision, although referrals were unaffected by COVID-19 training. These results suggest a need to enhance CHWs’ skills on managing suspected positive cases.

We found that CHWs in Bangladesh experienced a range of supportive mechanisms by government and nongovernmental actors, including training, supplies, and supervision, but these supports were not always uniformly distributed across cadres. While most CHWs were trained on COVID-19 prevention and referral either from the government or NGOs, HAs reported significantly more training than FWAs. Qualitative findings corroborate varying levels of training depending on the type of CHW. Our results show that being trained on COVID-19 predicted CHWs’ capacity to advise communities on symptoms and provide routine services. This finding aligns with other studies in which CHWs expressed the importance of training to them and the need for more training and capacity-building opportunities to retain a committed workforce, especially with the expanding and shifting roles during COVID-19.^6^^,^^22^^,^^23^ Nonuniformity of support provided to HAs and FWAs and differential cadre-specific effects on COVID-19 awareness building in the community and routine service provision merit attention in Bangladesh’s pluralistic community health system.

While CHWs experienced a range of supportive mechanisms by government and nongovernmental actors, these supports were not always uniformly distributed across cadres.

CHWs’ access to supplies was not uniform between the 2 cadres, and qualitative data indicated differing perspectives between policy and program stakeholders and CHWs about the adequacy of supply provision (by both the government and NGOs). The district in which the CHW was located partly accounted for this discrepancy in the availability of supplies. Additionally, nonaligned CHWs and policy/program stakeholders’ perspectives around supplies disbursed and received suggest a need for more responsive supply provision and oversight to ensure CHWs have the tools to adequately carry out their duties. Despite infection prevention being particularly crucial during disease outbreaks,^24^ our study confirmed the literature’s supply and (resupply) mechanism challenge,^25^^,^^26^ as both HAs and FWAs expressed limited and uneven access to infection prevention supplies and other tools (e.g., job aids) to perform their work. While access to supplies was not always guaranteed, existing supervisory structures enabled supportive supervision for both HAs and FWAs to continue their work throughout the pandemic through varied modalities. Our results, like other studies, indicate that mobile phones offer an adequate alternative for CHWs to access continued supervision in the context of COVID-19.^27^

Our study revealed that government support enabled CHWs in Bangladesh to sustain routine service provision in addition to taking on new responsibilities; however, CHWs experienced various COVID-19-related challenges, including increased workloads and unmet need for supplies. Government CHWs’ experiences were different from NGO CHWs because of their mechanisms of employment. Government-employed CHWs’ workload was increased because of an already-existing shortage of workers (before COVID-19) and extended responsibilities, such as participating in satellite clinics to arrange immunization sessions, implement a vitamin A campaign in the community, and act as a community skilled birth attendant, if trained. Both government- and NGO-employed CHWs receive 21 days of classroom training, but NGO CHWs receive 1–3 days of refresher training regularly,^28^ indicating a discrepancy in knowledge and skills that warrants attention. Additionally, NGO sector CHWs receive different equipment for their work such as bags, umbrellas, and notebooks, which CHWs did not mention in our study. Decreases in CHWs’ routine service provision were observed earlier in the pandemic across all health areas because of movement restrictions caused by lockdowns, lack of transportation, and community fear of COVID-19. The study found higher disruption regarding children’s immunizations than home visits for FP, which could be a reflection of constrained supply chains.^29^ Notably, some FWAs and HAs felt their ability to provide longer-term FP methods and vaccination, respectively, were limited because of a lack of clients coming to facilities/community health posts, reflecting global trends in decreased uptake in these health areas due to the pandemic.^29^^,^^30^ Our findings confirm that new responsibilities increased FWAs’ and HAs’ workload and detracted from routine service provision, exacerbating the ongoing challenge of a CHW workforce shortage in Bangladesh.^31^

CHWs in Bangladesh were able to sustain routine service provision, but they experienced various COVID-19-related challenges, including increased workloads and unmet need for supplies.

Qualitatively, CHWs expressed discontent with the lack of incentives accompanying the high expectations to work and undertake new responsibilities. This finding resonates with studies that show that CHWs require various forms of support, including training, supplies, and supportive supervision to effectively prepare them for their roles in service delivery, particularly when new knowledge or skills are required.^32^^,^^33^ Efforts to alleviate these shortages and provide CHWs performing new duties with proportional incentives may help boost morale and lessen the related negative consequences (i.e., increased workloads).^21^ Our findings align with outbreak studies that show CHW challenges in prioritizing routine responsibilities while adopting new ones without clear government guidance and support.^4^^,^^6^ Studies repeatedly emphasize that CHWs continue to provide routine services to a degree of normalcy when the rest of the health system becomes inaccessible for many, demonstrating resilience.^34^^,^^35^ Furthermore, communities place immense value on CHWs given that they are primed to provide emotional and psychosocial support during crises.^36^ Given the inherent trust that their communities place on them, government-employed CHWs in Bangladesh are in a critical position to educate and support COVID-19 immunization efforts,^22^ and future implementation research should involve and explore how CHWs link their communities to the government’s COVID-19 vaccination efforts.

Our study has several implications for future research and programming around COVID-19 and other outbreaks. Further programmatic efforts should be implemented to ensure equity in CHWs’ access to training opportunities and to increase opportunities for ongoing training to improve and sustain their routine and COVID-19-specific knowledge and skills.^1^^,^^37^ CHWs’ in-person supervisory preferences ought to be considered in developing safety, feedback, and reporting mechanisms in accordance with COVID-19 guidelines. To maximize the efficiency of CHWs and ensure their safety, further efforts to include CHWs’ needs in government-projected essential service and PPE (e.g., soap, hand sanitizer, and masks) distribution are crucial.^22^ Our findings advocate more attention and resources be devoted to coordinating efforts between governments and NGOs to ensure FWAs and HAs have equal access to critical supplies during the COVID-19 context and beyond.^23^^,^^24^ Although decreases in routine services were slight and improved over time during the pandemic, client-level effects were beyond the scope of this study; future research should explore the impact of routine service decreases on community health outcomes, particularly in the areas of FP and maternal and child health.

### Limitations

This study had some limitations. Our analysis focused solely on CHWs employed by the government sector in FP and maternal and child health areas. Other cadres of CHWs in Bangladesh are employed by the government as well as the NGO sector, including those working under the community clinic model, and they should be considered in future studies.^38^ Additionally, data collection occurred at one point in time in a dynamic pandemic context in which government-issues directives were frequently changing. Although we collected some information about these shifts, our results reflect the context in which the data were collected. Lastly, phone-based interviews were used as the method of data collection in this study because of the risks of in-person interviews, and this data collection modality may have elicited shorter responses and variable quality compared with in-person interview techniques.

## CONCLUSION

This study reinforces the important role that CHWs in Bangladesh play in the COVID-19 response in their communities and health facilities. Their demonstrated ability to carry out new responsibilities and maintain their routine duties to a great extent shows resilience, especially in the context of a global pandemic. Policy makers and program stakeholders alike must increase their efforts to support and sustain this critical workforce to effectively combat COVID-19 in their communities and reduce the effects of COVID-19 on population health.

## Notes

**Peer Reviewed**

**First published online**: August 23, 2022.
